# Migratory Metrics of Wound Healing: A Quantification Approach for *in vitro* Scratch Assays

**DOI:** 10.3389/fonc.2018.00633

**Published:** 2018-12-18

**Authors:** Sagar S. Varankar, Sharmila A. Bapat

**Affiliations:** National Centre for Cell Science, Savitribai Phule Pune University, Pune, India

**Keywords:** cell migration, live cell imaging, displacement, velocity, nearest neighbors, migratory modalities, fiji

## Abstract

Metastatic dissemination generates an aggressive disease facilitated by enhanced migratory and invasive properties. Experimental approaches employ several *in vitro* and *in vivo* assays toward quantification of these functionalities. *In vitro* assessments of cell motility often employ endpoint assays that rely on the global efficacy of wound closure and thwart quantification of migratory phenotypes observed during metastatic dissemination. Recent studies highlight the distinct signatures associated with individual vs. collective cell migration and necessitate the incorporation of these modalities into routine analyses. Advances in live cell imaging that permit real-time visualization of pathophysiological processes can be employed toward elucidating phenotypic plasticity associated with cell migration to overcome caveats inherent to end-point assays. Herein, we corroborate live cell imaging with the *in vitro* scratch assay toward quantification of migratory modalities in transformed cells. Our protocol describes a step-by-step approach for live cell setup of the scratch assay, and details analyses employed toward definition of three quantitative metrics viz., displacement, velocity and number of nearest neighbors. The current protocol (from scratch induction to data acquisition) is implemented for ~30 h and provides global/single-cell resolution of migratory phenotypes as opposed to the endpoint assays. Routine application of this protocol in cancer biology can aid the design of therapeutic regimes targeting specific migratory modalities and significantly contribute to the dissection of associated molecular networks.

## Introduction

Metastases represent an array of entropic events that facilitate disintegration of tissue architecture via acquisition of migratory/invasive properties. Cell migration, a crucial process in metazoan development, is often dysregulated under pathological conditions like fibrosis or cancer and contributes to disease associated morbidity. Maintenance of tissue homeostasis involves cross-talk of intrinsic cellular capacities with spatio-temporal cues in governing the onset, extent and mode of cell migration (1). Observations across several model systems identify two broad migratory modalities *viz*., individual or collective cell migration (CCM), wherein variations across physiological contexts are duly noted (2, 3). Activation of epithelial to mesenchymal transition (EMT) during wound healing and immune cell homing exemplify individual cell migration (4, 5); EMT gene signatures often correlate with higher efficacy of metastasis (6). The contributions of collectively migrating cells, have been captured during limb development and wound healing of tissues, and also depicted by clusters of circulating tumor cells. Co-ordination between both modalities is well-documented during gastrulation wherein collective migration induces the primitive streak followed by generation of the ectoderm by individually migrating cells (7).

Most studies on cell migration employ endpoint assays wherein data acquisition over large time intervals and analyses following assay termination ascertain the extent of migration (8). Inadvertently, these approaches associate wound healing efficacies with EMT and project individual cell migration as a dominant (if not only) process associated with metastasis with contributions of collective cell migration/proliferation being largely disregarded. These limitations also result in the failure to capture dynamics of migratory mode switching in response to extrinsic cues despite the drastically differential molecular signatures of CCM and EMT. Disparities in associating wound healing efficacies with cell morphology may arise due to the poor resolution of endpoint methods that can be surpassed by employing real-time imaging approaches. Time lapse microscopy provides enhanced resolution of cellular processes and allows the dissection of heterogeneous functionalities inherent to a system. Application of time lapse microscopy to traditional wound closure assays can effectively quantify and generate metrics for defining migratory modalities.

The present report is an approach toward the detection of migratory modes *in vitro* and relies on application and quantitation of time-lapse microscopy during the *in vitro* scratch assay. Data derived from such an experimental setup is processed to define individual cells as particles using the CLAHE and Threshold Plugins in Fiji followed by extraction of positional co-ordinates with the MTrack2 Plugin. Simultaneously, processed images are subjected to the BioVoxxel Plugin to identify the frequency of “nearest neighbours” in the field of focus. Positional co-ordinates are then utilized to calculate cell “displacement” and “velocity.” These metrics can be analyzed separately or in unison (Principal component analysis—PCA) to define discrete migratory modalities of cells.

## Materials and Equipment

### Reagents

#### Cell Lines and Medium

- Cell culture samples (e.g., mammalian differentiated cells, stem cells, primary cells, engineered cells, etc.). In this study, high grade serous ovarian adenocarcinoma (HGSC) cell lines A4 (9), OVCA420 (TRI, Australia) and OVCAR3 (NCCS Cell Repository) were used. The protocol has been verified with other HGSC (10) and breast cancer cell lines; non-cancerous cell lines—MDCK, HEK. **CAUTION**. Cell lines used for research purposes should be regularly authenticated and verified for mycoplasma infection.
- Minimal Essential Medium (MEM; Gibco, cat. no. 11095080)
- RPMI 1640 Medium (Gibco, cat. No. 11875135)
- 0.5% Trypsin-EDTA (TE; Gibco, cat. no. 15400054)
- Trypsin Phoshphate Versene Glucose (TPVG; HiMedia, cat. no. TCL031)
- Fetal Bovine Serum (FBS; MP Biomedicals cat no. 092910154)
- 100X MEM Non-essential Amino Acids Solution (NEAA; Gibco, cat. no. 11140050)

#### Buffers and Additives

- Mitomycin “C” (Sigma-Aldrich, cat. no. M4287). **CAUTION**. Mitomycin “C” can cause acute toxicity if inhaled, swallowed or exposed to bare skin and results in respiratory sensitization, germ cell mutagenicity, carcinogenicity, and reproductive toxicity; use of PPE is recommended.
- TGFβ1 (Thermo Fisher Scientific, cat. no. PHG9204)
- BMP7 (Thermo Fisher Scientific, cat. no. PHC7204)
- Paclitaxel (Sigma-Aldrich cat. no. T7402). **CAUTION**. Paclitaxel can cause respiratory sensitization, germ cell mutagenicity, carcinogenicity, and reproductive toxicity; use of PPE is recommended.
- Sodium chloride (NaCl; Fisher Scientific, cat. no. 15915)
- Potassium chloride (KCl; Qualigens, cat. no. 13305)
- di-Sodium hydrogen phosphate (Na_2_HPO_4;_ Merck, cat. no. 17951)
- Potassium di-hydrogen phosphate (KH_2_PO_4;_ Qualigens, cat. no. 19465)
- Dimethyl sulfoxide (DMSO; Sigma, cat. no. D-2650)

### Equipment

#### General Consumables and Equipment

- T-25 Tissue Culture Flask (Corning, cat. no. CLS430639)
- 50-mL centrifuge tubes (BD Falcon, cat. no. 352070)
- 15-mL centrifuge tubes (BD Falcon, cat. no. 352096)
- 1.5-mL micro-centrifuge tubes (Axygen, cat. no. MCT-175-C)
- Centrifuge for 15- and 50-mL centrifuge tubes (Hermle, cat. no. Z323K)
- Benchtop micro centrifuge for 1.5-mL centrifuge tubes (Eppendorf, cat. no. 5415R)
- CO_2_ incubator (37°C) (Thermo Scientific, cat. no. 4141)
- Biosafety cabinet (Kirloskar Electrodyne)
- Neubauer Chamber (Rohem, cat no. BS 748)
- Micropipette set
- 20-μl Micropipette tips (Axygen, cat. no. TF-300)
- 200-μl Micropipette tips (Axygen, cat. no. TF-200)
- 1,000-μl Micropipette tips (Axygen, cat. no. TF-1000)
- Autoclave
- Autoclaved plastic tip boxes
- 10-ml Syringe (Dispovan)
- 0.22-μm Millex-GP Syringe Filter Unit (Merck, cat. no. SLGP033RS)
- 15-ml Falcon tube rack
- 1.5-ml Eppendorf tube rack
- Tissue Culture treated 24 well-plates (Corning Costar, cat. no. CLS3527 SIGMA)
- Box with ice
- Rubber band
- Facial tissues

#### Live Cell Imaging Setup and Analysis

- Confocal laser scanning microscope (Leica, cat. no. TCS SP5)
- Leica Application Suite Advanced Fluorescence (Leica)
- Matlab r2013b (Mathworks, https://in.mathworks.com/)
- Fiji (Image J, Open source)

### Reagents Setup

#### Media Preparation

For A4 cells, combine 94 mL of MEM with 5 mL FBS (5% vol/vol) and 1 mL 100X NEAA (1X vol/vol) to prepare PA1 medium. For OVCAR3 and OVCA420 cells, combine 90 mL of RPMI 1640 with 10 mL FBS (10%vol/vol). **CRITICAL**. All steps to be followed under aseptic conditions in a biosafety cabinet. Prior to use, FBS should be filtered using a 0.22 μ filter. The constituted media solution should be stored at 4°C for no more than 2 weeks.

#### Drug and Additive Preparation

0.5 mg/mL of stock solution for mitomycin'C' was prepared in sterile distilled water. 5 μM stocks of paclitaxel were prepared in 100% DMSO. TGFβ1 and BMP7 were reconstituted at 20 μg/mL in sterile 4 mM HCl containing 1 mg/mL bovine serum albumin. **CRITICAL**. All steps to be followed under aseptic conditions in a biosafety cabinet. The reconstituted drugs and additives can be stored at −80°C for a year and −20°C for 6 months.

#### 1X PBS (pH 7.40) Preparation

For 1 L of sterile 1X PBS preparation add 8 g NaCl (137 mM), 0.2g KCl (27 mM), 1.44 g Na_2_HPO_4_ (100 mM) and 0.24 g KH_2_PO_4_ (1.8 mM) to 800 mL of distilled water. Adjust pH to 7.4. Add distilled water to make up volume to 1L. Sterilize solution by autoclaving at 121°C 15psi for 20 min.

### Equipment Setup

#### CO_2_ Incubator (37°C)

Culture cell lines in a 5% CO_2_ atmosphere at 37°C. **CRITICAL**. Incubation criteria may change as per the systems under study.

#### Live Cell Imaging Microscope Setup

Live cell imaging is performed at 37°C in a 5% CO_2_ atmosphere. **CRITICAL**. Incubation criteria may change as per the systems under study. Prior to live cell setup allow the temperature and CO_2_ to set at 37°C and 5%, respectively in the Leica TCS SP5 microscope. Configure stage settings to ensure proper position setup during live cell setup. Ensure proper pH maintenance in the culture medium before and during live imaging setup.

### Step-Wise Procedures

**CRITICAL**. Appropriate biosafety procedures need to be followed throughout the assay setup. Steps 1–5 should be performed in a biosafety cabinet.

**Passaging of cells** [TIMING ~15 min per cell line]

1) Harvest sub-confluent, growing A4 cells from a T-25 tissue culture flask by washing cells twice with sterile PBS, adding 500 μL 0.5% TE solution and incubating at 37°C for 2 min. After the incubation, add equal volume of PA1 medium to the flask and gently tap the flask to disperse the cell monolayer. Take an aliquot from the cell suspension and obtain cell counts using a Neubauer Chamber. Follow a similar protocol for OVCAR3 and OVCA420 cells; substituting TPVG for TE, reducing TPVG incubation to 1 min at 37°C and PA1 with RPMI + 10% FBS medium.
  **CRITICAL STEP**. Incubation criteria following trypsin addition varies as per the cell systems under study and must be optimized to ensure optimal monolayer dispersal and minimal loss of cell viability.
2) Seed cells into a 24-well-tissue culture plate to obtain a confluent monolayer, following a 24 h incubation at 37°C. This allows proper adherence and spreading of cells on the substrate. The required number of cells for a confluent monolayer depends on cell type, size of culture plate, cell doubling time and needs to be adjusted properly.
  **CRITICAL STEP**. Maintain a constant seeding number specific to each cell line; this ensures reproducibility of results.
  **?** Troubleshooting
  PAUSE POINT Harvested cells can be kept on ice for 1 h.

**Scratch or wound induction** [TIMING−15 min]

3) After 24 h incubation wash the cell monolayer with sterile 1X PBS and replace with serum free medium. Incubate the cells at 37°C for 24 h to acclimatize the monolayer to serum free conditions.
  **CRITICAL STEP**. Serum deprivation ensures removal of undefined growth factors, thus ensuring a specific response to wounding and other extrinsic stimuli.
  **CRITICAL STEP**. Complete serum deprivation may affect viability in some cells, hence optimization may be necessary.
4) Scrape the cell monolayer in a straight line with a 200 μL pipette tip and remove debris by washing with 500 μL of sterile 1X PBS. Replace with serum free medium containing mitomycin “C” (10 μg/mL). If the study includes effect of specific growth factors or other compounds, they should be included in the serum free medium.
  **CRITICAL STEP**. It is important to create wounds of similar widths to ensure comparison across samples.
  **CRITICAL STEP**. Washes with PBS need to be gentle with minimal disturbance to the wound edge.
  **CAUTION**. Cell lines with extensive cell—cell contacts exhibit a tendency to peel from cell monolayer following scratch induction, if not handled gently.
  **CRITICAL STEP**. Mitomycin “C” serves as a proliferation inhibitor to eliminate the contribution of cell division to wound closure. Optimal mitomycin “C” treatment ensures minimal loss of viability with maximum inhibition of cell division.

**Data acquisition** [TIMING−24h]

**CRITICAL STEP**. In this protocol, images are captured with a Leica TCS SP5 scanning confocal fluorescence microscope. Depending on the equipment availability, other microscopes equipped for time lapse microscopy can be used to collect similar images.
5) Setup 24 well-plate in a CO_2_ chamber on the microscopic stage for imaging under a 10X objective lens. Define exact positions and focal planes for the scratch area in each well and set an imaging interval of 30 min over a span of 24 h. Images will be captured at regular intervals and the data will eventually be saved as a .lif file. Export time lapse data as .avi /.mov files for analysis.
  **CRITICAL STEP**. Prior to the setup of time lapse microscopy, switch on the CO_2_ and temperature equilibration system atleast 30 min in advance to ensure that the parameters are set at 5% CO_2_ and 37°C, respectively.
  **CRITICAL STEP**. Use of chamber slides and coverslip bottom plates can allow the use of higher magnification and better resolution of migrating parameters.
  **CRITICAL STEP**. Fluorescently labeled cells can be used for this assay to improve resolution of images.
  **?** Troubleshooting
  PAUSE POINT Data processing can be conducted as per convenience of user.

**Data analysis** [TIMING ~15 min per file]

**Figure 1:**
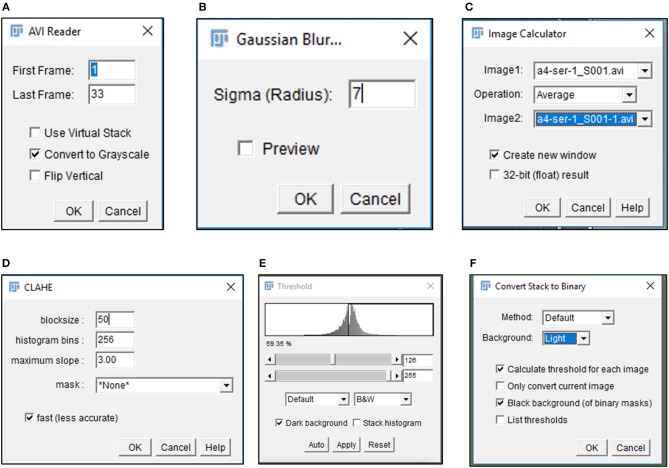
Image processing of time lapse data. Pop-up windows generated during image processing include the **(A)** import of data as a gray-scale image, **(B)** selection of appropriate sigma radius for a Gaussian blur, **(C)** illumination correction with Image calculator, **(D)** CLAHE assisted contrast enhancement, and **(E,F)** definition of particles by Threshold tool.

**Figure 2:**
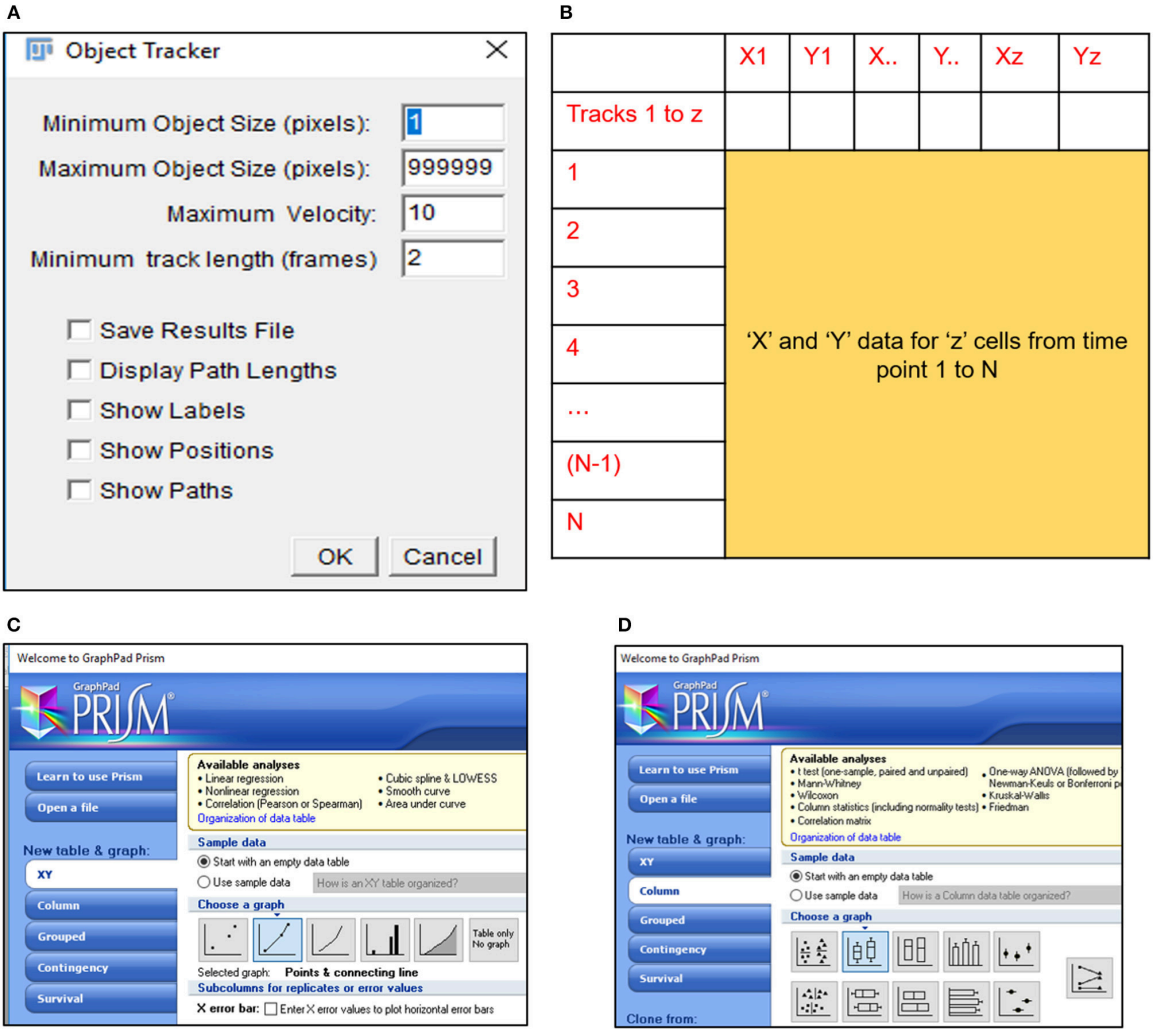
MTrack2 assisted extraction of migratory data. **(A)** MTrack2 plugin can be applied to post-threshold images to extract the positional co-ordinates for particles based on size and velocity; **(B)** Schematic representation of array arrangement used for calculation of cell displacement and velocity; Schematic representation of Graphpad Prism 5 tools used for representing **(C)** displacement trajectories and **(D)** velocity.

**Figure 3:**
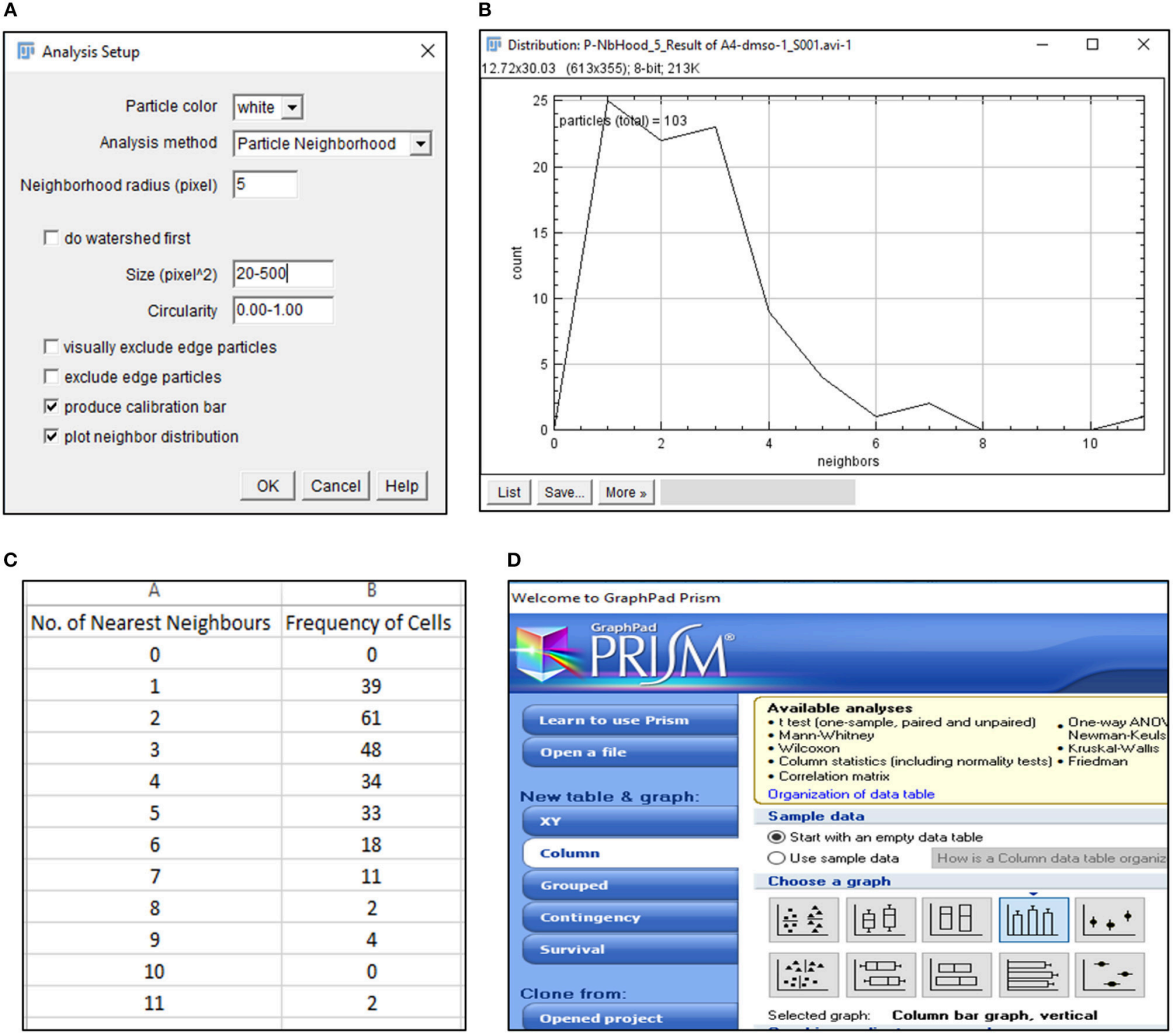
Detection of nearest neighbors with BioVoxxel plugin. **(A)** BioVoxxel plugin application to post-threshold images extracts the nearest neighbors in particle vicinity based on definition of neighbor radius, particle size and circularity by the user; **(B)** Representative graph generated by BioVoxxel plugin to depict the frequency of nearest neighbors; Data from this file can be exported as.csv file **(C)**; **(D)** Schematic representation of Graphpad Prism 5 tools used for representing frequency of nearest neighbors.

**Figure 4:**
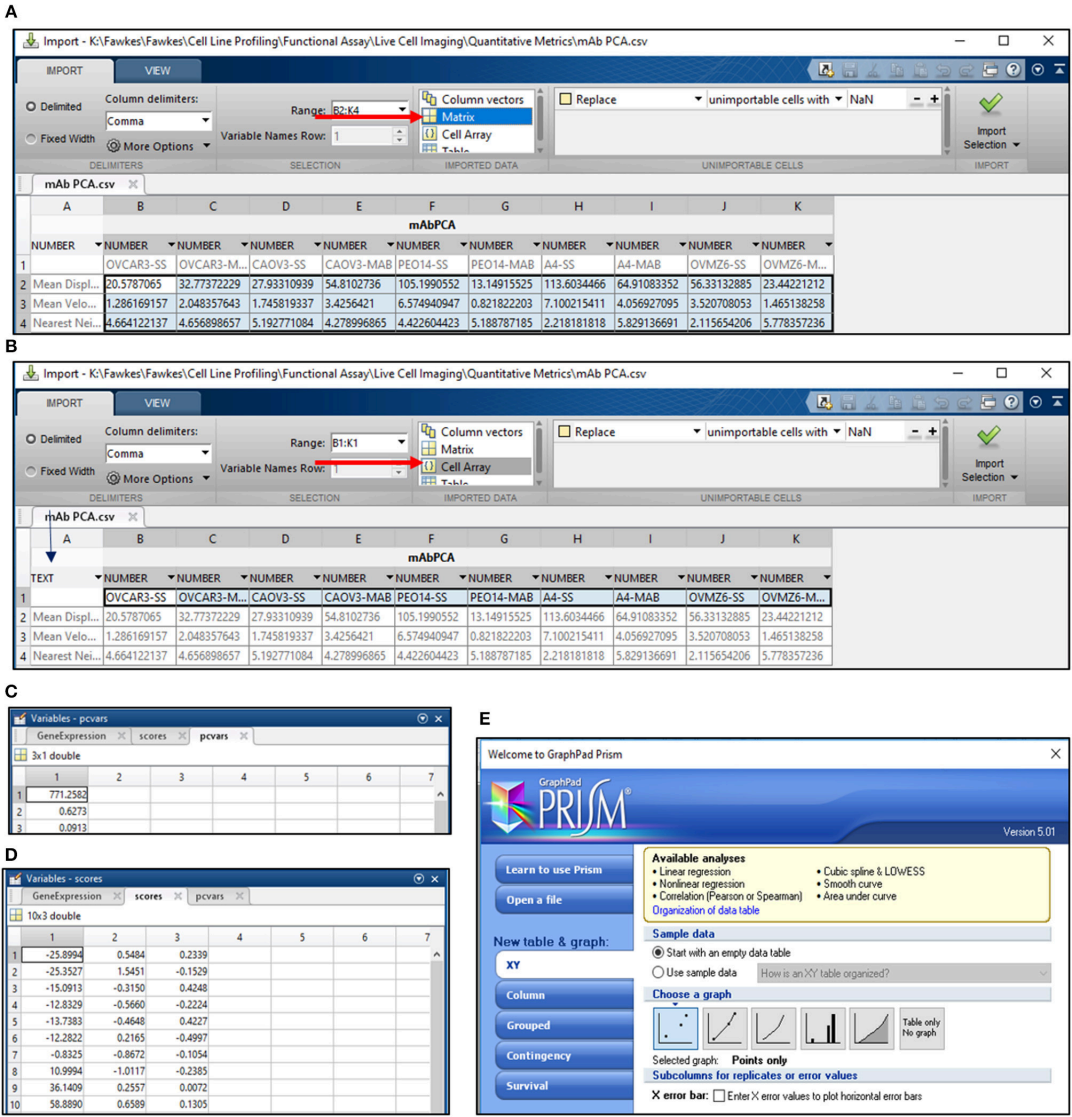
PCA analysis in MATLAB. **(A)** PCA analysis in MATLAB involves the import of data from a.csv file wherein the data is arranged as depicted. Import of data involves the selection of cells containing values for the migration metric and their import as a matrix (arrow in red); **(B)** Sample names are imported by selection of cells containing sample labels and conversion to text (blue arrow). The labels are imported as a cell array (red arrow); Output for variance **(C)** and scores for PC1 and PC2 **(D)** are obtained following execution of the command line; **(E)** Schematic representation of Graphpad Prism 5 tools used for representing PC1 and PC2 scores obtained from the analysis in MATLAB.

**Figure 5:**
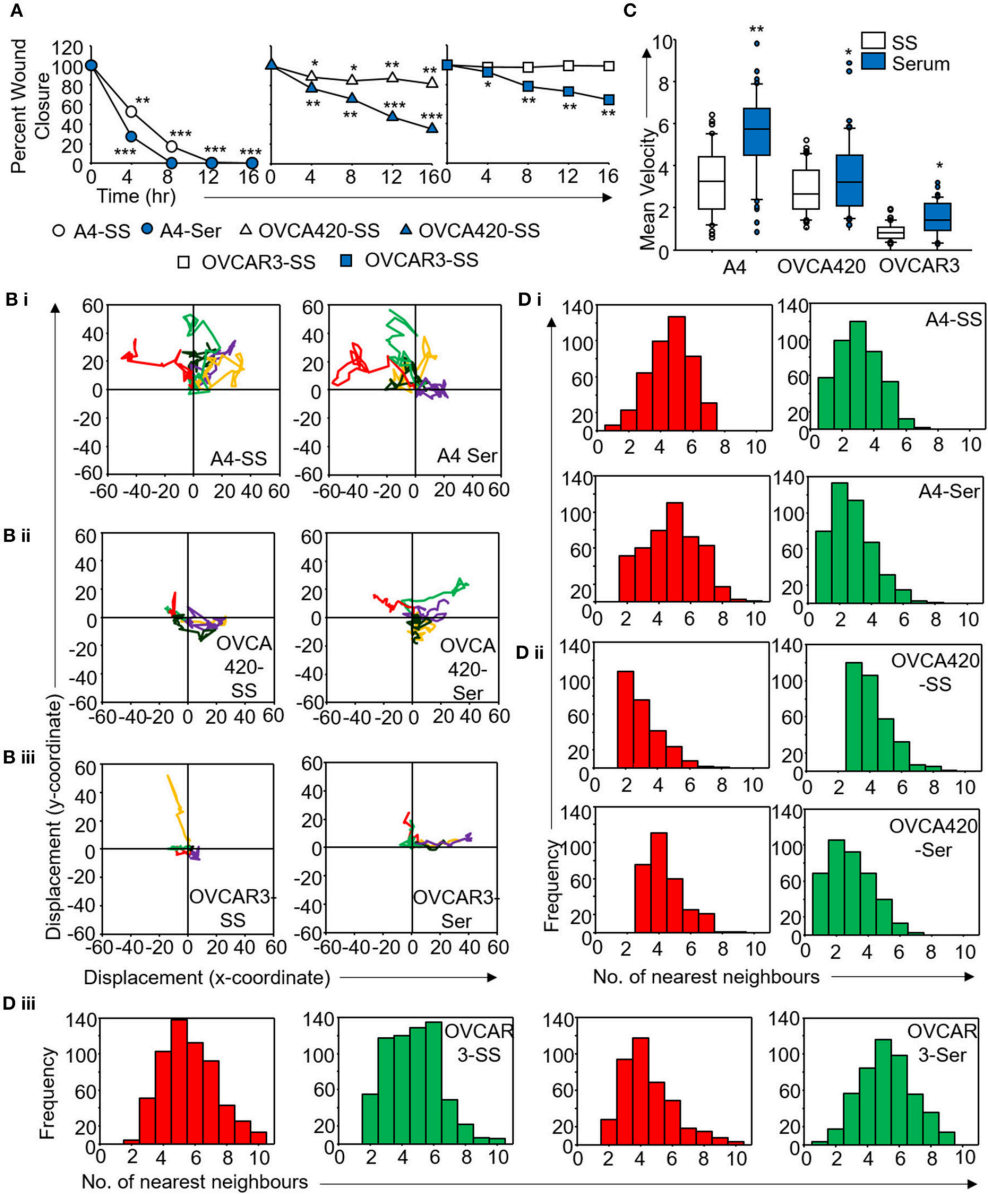
Derivation of quantitative metrics for migration in ovarian cancer cell lines. **(A)** Percent wound closure derived from *in vitro* scratch assays for A4, OVCA420, and OVCAR3 cells in the absence (SS) and presence of serum (Ser); **(B)** Trajectories depicting direction of migration for **(i)** A4, **(ii)** OVCA420, and **(iii)** OVCAR3 cells derived from : “x” and “y” positional co-ordinates over a 16 h duration of live cell imaging.; **(C)** Representative boxplots depicting mean migratory velocity for A4, OVCA420, and OVCAR3 cells; **(D)** Frequency of nearest neighbors for **(i)** A4, **(ii)** OVCA420, and **(iii)** OVCAR3 cells at 0 h (red) and 16 h (green) time points. Experiments were performed in the absence and presence of serum and altered migratory metrics were duly noted. All data are representative of experiments performed in triplicate and are depicted as mean ±SD, *p < 0.05, **p < 0.01, ***p < 0.001. Details of PCA values are provided in Table S2.

**CRITICAL STEP**. Uneven illumination of the image field must be corrected in ImageJ or other related softwares providing the same options.
6) Import .avi files in Fiji software (https://imagej.net/Fiji/Downloads) and select the “Convert to Grayscale” option in the pop-up window (Figure 1A, Figure S1A).
  **CRITICAL STEP**. Avoid selection of “Virtual Stack” option in the pop-up window during image import to prevent errors in application of processing plug-ins required in subsequent steps.
  **CAUTION**. .avi files imported for image analysis should not have time or resolution scales overlaid on to them. Pixel contributed by these scale bars interfere with subsequent stages of processing and image analysis.
7) Perform an illumination correction of the imported image by generating an inverted duplicate of the file (Figure S1B). Select the duplicate image and apply the “Gaussian Blur” filter to perturb image sharpness, such that cell edges are visible as silhouettes (Figure 1B, Figure S1C). Select the “Image Calculator” tool to derive an averaged image from the original and inverted file (Figure 1C, Figure S1D).
  **CAUTION**. Image duplication must include an import of all stacks representing different time points of data.
  **CAUTION**. Gaussian blur adjustments depend on the sharpness of the image and need to be adjusted such that illumination correction results in minimal loss of data.
8) Subject the corrected image to the CLAHE plugin to improve the overall contrast of the image for particle analysis (Figure 1D, Figure S1E). Optimal selection of block size in CLAHE results in a processed image with sharp cell edges and dense cytoplasm distinguishable from the culture dish surface.
  **CRITICAL STEP**. CLAHE plugin should be applied to ensure maximum improvement in contrast with minimal distortion of image quality.
9) Select “Threshold” function in Fiji to identify individual cells in the illumination and contrast adjusted image (Figure 1E). Image generated post “Threshold” application requires a light background to permit further processing (Figure 1F, Figure S1F).
10) Select the MTrack2 plugin to extract the X and Y co-ordinates of individual cells across time stacks in the image field (Figure 2A).
  **CRITICAL STEP**. Object size and maximum velocity definition in the MTrack2 plugin govern the detection of cells in this analysis.
  **CRITICAL STEP**. “X” and “Y” co-ordinates extracted with the MTrack2 plugin are saved as a.txt file labeled “trackresults”
  **CRITICAL STEP**. Plugins other than MTrack2 can be used for the analysis depending on the compatibility of the system.
11) Import “trackresults” file in Microsoft Excel 2013 (Figure S2A). The file is saved as a continuous array comprising of “X” and “Y” co-ordinates for all the particles (cells) detected by MTrack2 over the duration of the time lapse video. Data arrangement in the array is as depicted in Figure S2B.
Edit the file to ensure that co-ordinate data for all particles is presented as continuous rows (Figure 2B).
  **CRITICAL STEP**. Exclude incomplete migratory tracks and proliferating cells from the analysis to prevent inclusion of incomplete datasets (Figure S2C). Nuclear marker can be employed to identify proliferating cells.
12) Calculate displacement, squared distance and velocity for individual cells from the “X” and “Y” co-ordinates as follows.
  $$\begin{array}{l} X\;displacement\;\left(Xd\right)=\;Xt-X\left(t-1\right)\;-\text{Equation 1}\\ Y\;displacement\;\left(Yd\right)=Yt-Y\left(t-1\right)\;-\text{Equation 2}\\ Displacement\;\left(d\right)=\;\sqrt{{\left(Xd\right)}^{2}\;+\;{\left(Yd\right)}^{2}}-\text{Equation 3}\\ Squared\;Displacement\;\left(SD\right)=d^{2}-\text{Equation 4}\\ Mean\;Velocity\;\left(v\right)=\frac{1}{N}\;\sum_{t=0}^{T}\frac{SD}{t}\;-\text{Equation 5} \end{array}$$
  Where X and Y are cell position co-ordinates at different time points
  “t” is the time point at which cells have undergone a displacement “d”
  “T” is the final time point at which the experiment was terminated
  “N” is the total number of time points
  **CRITICAL STEP**. While “SD” has not been separately plotted in our analysis, its calculation is crucial for the derivation of Mean velocity. Similarly, cell movement has been depicted as “X_d_” and “Y_d_” in our graphical representations as opposed to “displacement” which has been used for calculating “SD” and eventually “v”
  **CRITICAL STEP**. The time span over which parameters are calculated can vary depending on the extent of wound healing performed by each cell. The analysis can also be performed over a mean time or different time points, respectively.
13) Export Displacement data to GraphPad Prism 5 and select a “Points and connecting line” graph to depict the displacement trajectory (Figure 2C). Edit the trajectory graph as per requirement.
  **CRITICIAL STEP**. Number of trajectories plotted on the displacement graph should be optimal to ensure minimal congestion and maximal detection of cell movement.
14) Export Velocity data to GraphPad Prism 5 and select a “Box and whiskers vertical” graph to depict the mean velocity of all cells under consideration (Figure 2D). Edit the velocity graph as per requirement.
  **CRITICAL STEP**. Data from equal number of cells must be plotted to ensure accurate comparisons.
15) Select BioVoxxel plugin to determine number of nearest neighbors (n) for each cell (Figure 3A).
  **CRITICAL STEP**. Particle size, circularity and pixel distance definition are crucial toward detection of nearest neighbors and require optimization based on cell types.
  **CRITICAL STEP**. Definition of particle size is critical in extraction of positional information. Pixel sizes must be adjusted to recognize each cell as a distinct particle.
  **?** Troubleshooting.
16) Save average nearest neighbors results as.txt file and open in Microsoft Excel 2013 (Figures 3B,C).
17) Export Nearest Neighbors (Nn) data to GraphPad Prism 5 and select a “Colum bar vertical” graph to depict the frequency of nearest neighbors of all cells under consideration (Figure 3D). Edit the graph as per requirement.
  **CRITICAL STEP**. Data from equal number of cells must be plotted to ensure accurate comparisons.
18) Calculate mean SD, velocity and Nn for the entire cell population under study and export it to a separate Workbook in Mircosoft Excel 2013. Save this file for PC analysis in MATLAB in the.csv format. Arrange the data as depicted in Figure 4A.
19) Open MATLAB r2013b and import the.csv file for PC analysis. Select all the data cells, label it as per requirement, for e.g., **Matrix Label** and import the selection as a “Matrix” (Figure 4A).
20) Select cells with sample labels and click on the “Cell array” option for export. Select “Text” format in the cell arrays and apply it to the entire selection. Label the Cell Array as “Samples” and import the selection (Figure 4B).
21) Open the command window in MATLAB and use the following script to obtain values for PC1 and PC2 (Figures 4C,D):
  >> [~,scores,pcvars] = princomp(**Matrix Label**); - provides PC values and variance scores
  >> x = scores (:,1);
  >> y = scores (:,2);—provides values for PC1 and PC2
22) Export values for PC1 and PC2 to GraphPad Prism 5 and select a “Points only” graph to depict PC data for all samples under consideration (Figure 4E). Edit the graph as per requirement.
23) Graphs representing each of these datasets are depicted in Figure 5. Significant differences following treatments in wound healing efficacies and mean velocity were calculated by paired Student's *t*-test using the Sigma Stat 3.5 Software. Statistical details of PCA are provided in Table S1 and Table S2.
  **?** Troubleshooting

## Timing

Steps 1–2: Passaging of cells: ~15 min per cell line

Steps 3–4: Scratch or wound induction: 15 min

Step 5: Data acquisition: 24 h

Steps 6–9: Data analysis: ~15 min per file.

## Anticipated Results

**Figure 6:**
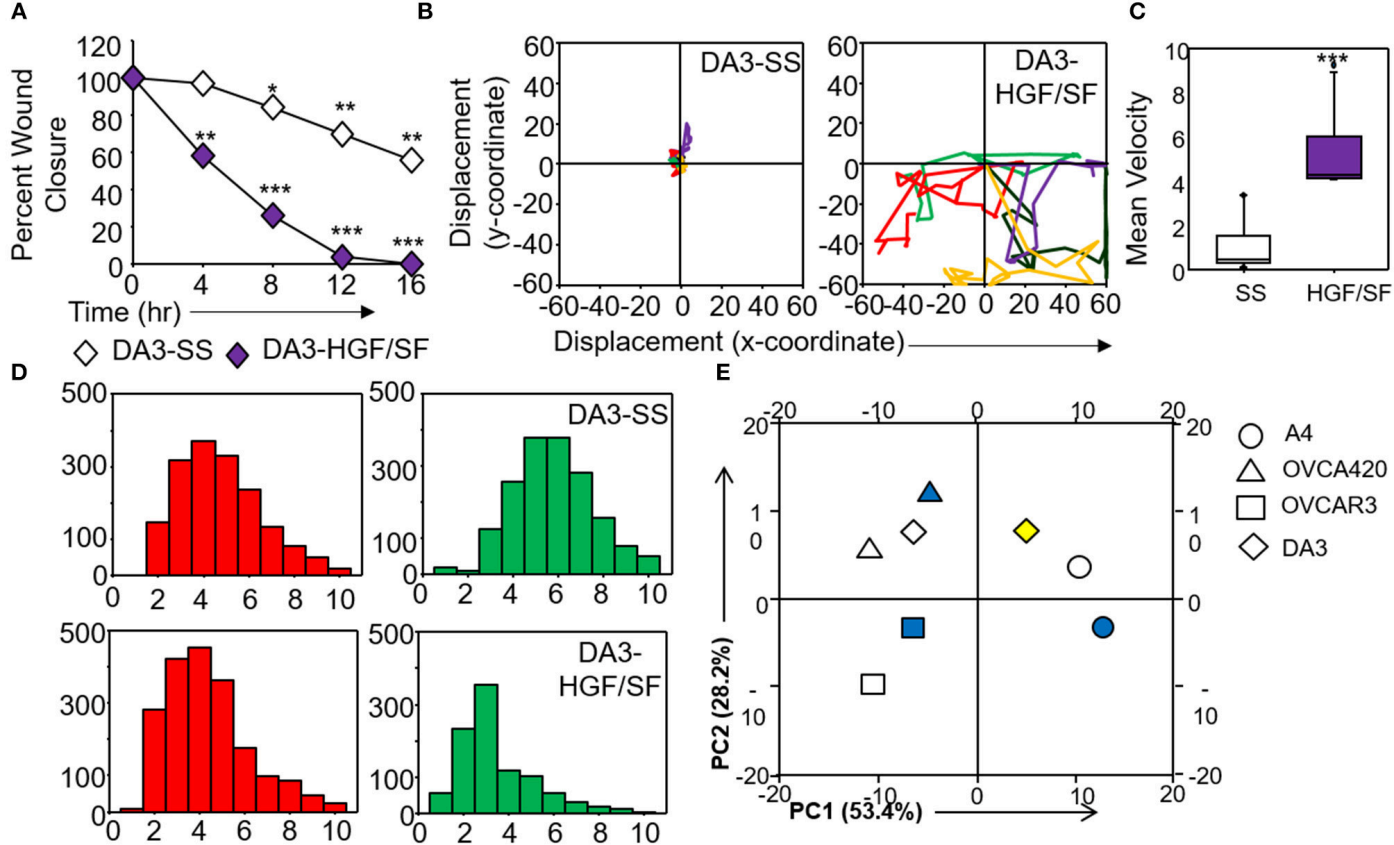
Validation and corroboration of quantitative metrics. **(A)** Percent wound closure derived from *in vitro* scratch assays for DA3 cells in the absence of serum (SS) and presence of HGF/SF; **(B)** Trajectories depicting direction of migration for DA3 cells derived from “x” and “y” positional co-ordinates over a 16 h duration of live cell imaging in the presence of HGF/SF as compared to control (SS); **(C)** Representative boxplots depicting mean migratory velocity for DA3 cells; **(D)** Frequency of nearest neighbors for DA3 cells at 0 h (red) and 16 h (green) time points. Alterations in migratory metrics were duly noted; **(E)** Principle component (PC) analysis of time-lapse imaging-based migration data of A4, OVCA420, OVCAR3, and DA3 cells, PC1—variance between displacement (Final Y) and velocity vs. nearest neighbors, PC2—variance between displacement and velocity, filled (blue) and empty shapes indicate presence and absence of serum, respectively; HGF/SF exposure is represented by filled yellow shape. All data are representative of experiments performed in triplicate and are depicted as mean ±SD, *p < 0.05, **p < 0.01, ***p < 0.001. Details of PCA values are provided in Table S2.

Application of time lapse microscopy to *in vitro* wound healing assays identifies migratory modalities and discerns phenotypic transitions during the process. Quantification of three migratory metrics viz., mean square displacement, velocity and number of nearest neighbors, can examine the effects of media components, additives, small molecules, and chemotherapeutic agents on migration modes with enhanced comprehension of physiological/diseased states. Sub-optimal results obtained from the protocol can be improved based on instructions provided in Table 1. Data representation relies on requirements of the study. Independent representation of migratory metrics as bar graphs can identify the physical parameter most affected in response to specific additives. Figure 5A depicts the information obtained for percent wound closure from a traditional scratch assay. Application of live cell imaging allows the calculation of individual cell trajectories (Figure 5B), quantification of average velocity (Figure 5C) and the effect on frequency of nearest neighbors (Figure 5D) as depicted for three ovarian cancer cell lines in the absence and presence of serum. As is evident from the data, serum addition enhances cell velocity and individual migration in A4 cells, whereas OVCA420 and OVCAR3 continue collective migration (Videos S1–S6). Figure 6 depicts the application of our analysis to a publicly available live cell imaging based scratch assay for DA3 cells and correlates with observed results. Principal component analysis (PCA, Figure 6E) of the quantitative metrics for A4, OVCA420, OVCAR3, and DA3 cells depict the segregation of migratory phenotypes in presence of different media conditions. It is noteworthy that a PCA plot allows concise representation of this multi-dimensional data to improve interpretation when comparing multiple model systems. Corroboration of three metrics allows delineation of three migratory phenotypes passive CCM (pCCM), active CCM (aCCM) and EMT as mentioned in Table 2. Our analysis captures serum induced changes in cells; in A4, a switch from aCCM+EMT to solely EMT mediated migration is evident. Application of this analysis to our cell systems in the presence of EMT inducer TGFβ results in a shift toward the EMT phenotype in OVCA420 and A4 but not in OVCAR3 cells (Figure S3; Videos S7–S9). Interestingly, our analyses highlights that A4 cells induced with TGFβ exhibit single cell intrusion into the wound area depicting a pure mesenchymal phenotype. However, the overall migratory rate of these cells in the absence of serum and presence of TGFβ and mitomycin “C,” implicates (1) contributions of pro-proliferative role of TGFβ in ovarian cell wound healing and (2) higher efficacy of wound closure in partial EMT cells that exhibit aCCM+EMT mediated migration over purely mesenchymal cells that lack directionality in migration. These findings further strengthen the need of a real-time analysis for migratory modes, as end-point assays often correlate these intruding single cells with efficient wound healing. Regardless of the representation applied, this analysis provides a higher resolution of migratory phenotypes in cell systems and holds greater application to disease biology as opposed to endpoint assays.

**Table 1:** Troubleshooting table (?).

**Step**	**Problem**	**Possible reason**	**Solution**
2	Low cell attachment after passaging	Over-trypsinization of cells	•Avoid over-trypsinization of cells •Trypsin incubation should be optimized for cell lines to ensure maximum viability
5	Extensive cell death observed during live cell migration	•Optimal growth conditions of temperature and CO_2_ were not maintained •Toxic concentration of dyes or fluorochromes used for cell detection	•Test CO_2_ levels and temperature settings in microscope chamber prior to live cell setup •Affix the CO_2_ chamber onto the tissue culture plate to ensure minimum leakage •Prior to time lapse setup, optimize concentration of dyes/fluorochromes used
7	Fiji unable to differentiate cells from background in particle definition	Shadow effects in images used for processing	Ensure imaging setup with minimal light associated variations in the focus field
		Sub-optimal contrast adjustment with CLAHE	Enhance local contrast for each image to distinguish cells from background. Contrast adjustment for data representation and particle analysis may vary
7	Derived metric values do not correlate with observed live cell data	Sub-optimal selection of pixel size for particle definition	Pixel size selection should be optimized for each cell line to appropriately extract positional information

**Table 2:** Quantitative metrics derived migratory phenotypes.

	Passive CCM* (pCCM)	**Active CCM (aCCM)**	EMT*
Displacement (d)	Low	Moderate	Low
Velocity (v)	Low (v <2)	Moderate (2 <v <4)	High (v>4)
No. of Nearest Neighbors (Nn)	High (Nn>3)	High (Nn>3)	Low (Nn <3)

*CCM, collective cell migration; EMT, Epithelial to mesenchymal transition. Details of statistical analyses are provided in Table S1*.

## Discussion

Combination of time lapse microscopy with the *in vitro* scratch assay defines quantifiable metrics for distinguishing individual vs. collective migratory modes across cell systems. Widespread application of *in vitro* scratch assay and its adaptations stem from the inherent simplicity in execution and analysis; however poor resolution of migratory modalities in these assays raise concerns pertaining to inferences derived toward examining pathological conditions (10–12). Moreover, targetable molecular players contributing to distinct modalities vary drastically despite comparable observations of wound closure efficacies (6, 13). Our protocol, thus enhances the resolution of *in vitro* scratch assays in routine application in cancer biology. Analyzing a publicly available dataset of DA3 cells further highlights the broad applications of our approach (14).

Recent application of quantitative phase imaging measured proliferation potential, movement and morphology of seeded cells thus defining *in vitro* heterogeneity based on differences in cell area and volume, however metrics capable of distinguishing individual vs. clustered cells were not discerned in this study (15). Similarly, particle image velocimetry aided dissection of collectively migrating cells identified bursts of activity at wound edge (16). However, existence of strong cellular contacts in the cell systems prevented phenotypic transitions thus, limiting the analysis of individual cell migration. Separately, time lapse microscopy mediated quantification has also identified migratory efficacies in response to durotaxis and associated molecular regulators by coalescing velocity and traction force measurements (17). While both studies exclusively quantify collective cell migration, complementation of these approaches with our analysis may enhance their applications to disease biology by quantifying transitions in migratory modes (10).

Previously, exhaustive derivation of quantitative metrics utilized a micropillar array to study wound edge transitions by comparison with a binary-mixture solidification model (18). Six quantitative metrics derived in this study and the precise fabrication of an array enhanced the accuracy and reproducibility of results. However, the array design constantly disrupts cell contact, a relatively stochastic process *in vivo* and also requires extremely specialized tools for study as opposed to our assay. In previous studies, extensive application of particle imaging velocimetry to derive Finite Time Lyapunov Exponents (FTLE) scalars representing the stretching of a trajectory provided directional cues to quantify migration in response to altered micro-environmental conditions (19). Increased FTLE was associated with enhanced disorder which essentially occurs during metastasis associated migration, besides achieving cell trajectory based demarcation of collectively and individually migrating cells which is comparable to frequency of nearest neighbors (Nn) employed in our analysis.

Quantification of migratory metrics can be routinely applied along with scratch assays to dissect the modality of migration in response to growth factors, chemotherapeutic agents, nutrient depletion, extra-cellular matrix components, and gene manipulation protocols (10). We have applied these metrics across cancer and non-transformed cell lines, thus expanding the spectrum of this method. Moreover, assessing differential activation of migratory modes across cell types can establish functional correlations with pathological conditions. These quantitative metrics can also be applied to decipher the migratory capabilities of sorted tumor populations to assess heterogeneity and may prove crucial in designing metastasis associated therapeutic regimes. Stable tagging of cells with fluorescent lipophilic dyes or protein can enhance the resolution of live cell imaging, besides providing the additional metric of label intensity. Promoter directed switching of fluorescence can also detect phenotypic changes associated with migration and decipher critical molecular cues. Similarly, labeling of cytoskeletal components can capture rearrangements associated with cellular morphology/shape in real time migration. Viable nuclear labeling will allow the inclusion/exclusion of proliferative potential during wound closure analysis.

While the assay dramatically enhances outputs obtained from the scratch assay we accept obvious limitations which can be overcome with specific adjustments. Our study employed a relatively homogeneous system of cell lines whereas utility of this assay toward physiological assessment of cell migration would require the development of co-culture systems, to assess the influence of heterotypic cell populations on migration. Similarly, due to the use of a 2-D system, the effects of tissue architecture on cellular migration cannot be assessed in our systems. Appropriate modifications and coalescence of biophysical approaches may aid in improving this analysis and application to 3D models and developmental stages. This protocol can be extensively employed for assessing migratory potential of adherent cells, the migratory potential of cell suspensions by virtue of active fluid displacement cannot be discerned.

Our study thus provides a detailed protocol for the incorporation of time lapse microscopy into cell migration assays and its adoption for analysis of publicly available datasets. The enhanced resolution provided by this system allows detection of phenotypic plasticity inherent to biological systems and generates quantitative metrics capable of discerning switches in cellular functionalities in response to extrinsic or intrinsic cues. We believe the incorporation of these analyses toward development of relevant imaging tools and in drug development pipelines may provide a directed targeting of metastatic phenotypes and address the heterogeneity associated with tumor metastatic dissemination.

## Author Contributions

SV and SAB contributed to the project design and writing of manuscript. SV contributed to execution of experiments and analysis of results. All authors critically revised and edited the manuscript and approved the final draft.

### Conflict of Interest Statement

The authors declare that the research was conducted in the absence of any commercial or financial relationships that could be construed as a potential conflict of interest.
