# Supplementary material for: Migratory Metrics of Wound Healing: A Quantification Approach for in vitro Scratch Assays
Source: Front Oncol. 2018 Dec 18;8:633. doi: 10.3389/fonc.2018.00633 (PMC6305394; doi:10.3389/fonc.2018.00633)
Supplement: Supplementary file 10 [file Data_Sheet_1.PDF]

Supplementary Information

FIGURE S1

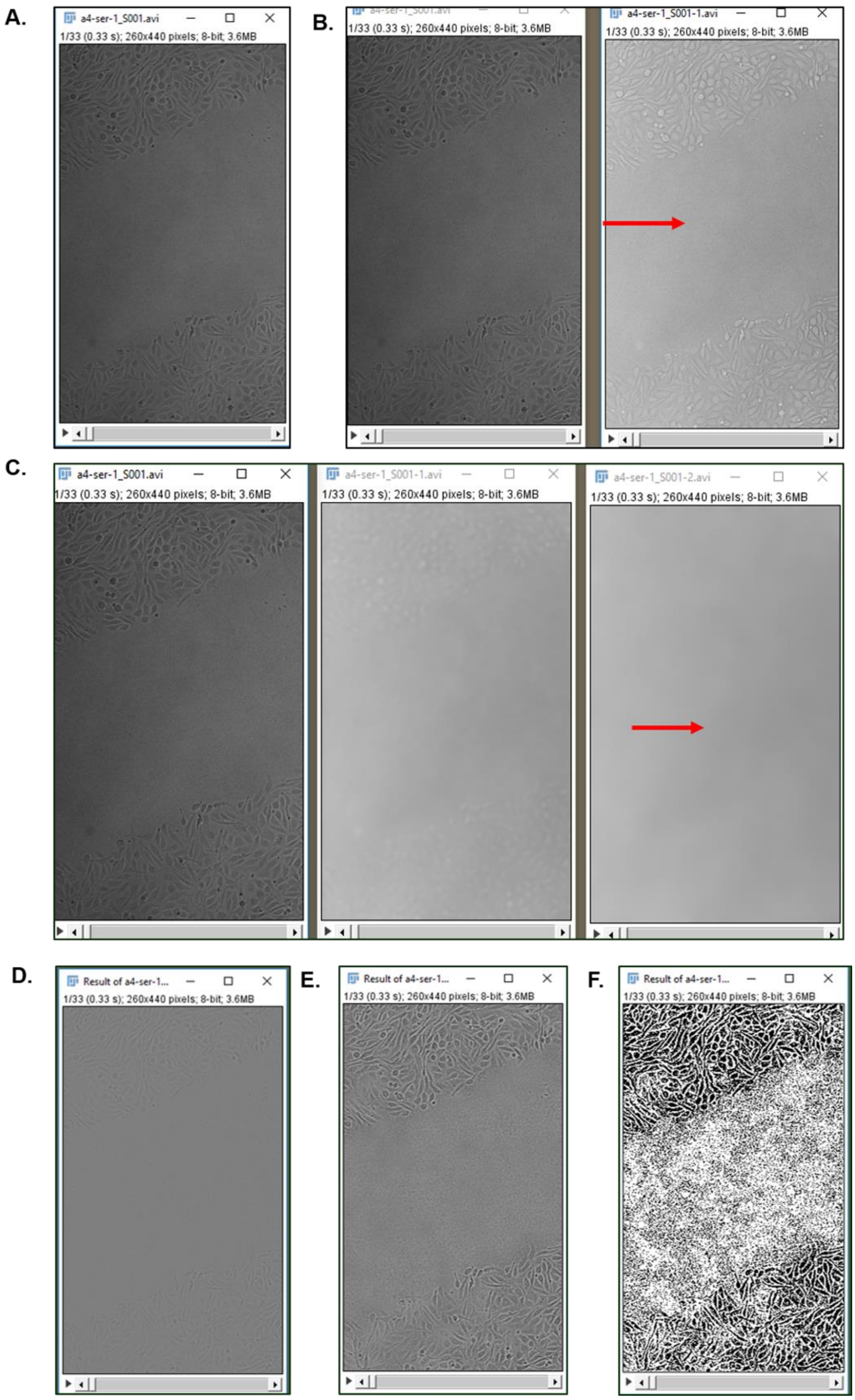

FIGURE S1. Image processing with Fiji. A. Image imported from .avi file in Fiji; B. Duplicated and inverted image for illumination correction. Inverted image is indicated by a red arrow; C. Application of optimal spatial radius through the 'Gaussian Blur' tool to permit illumination correction. The incorrect 'Gaussian Blur' is indicated by red arrows; D. Image obtained *via* application of the 'Image Calculator tool'. The original and inverted image data is averaged with

this tool; E. Application of CLAHE to the illumination corrected image improves contrast of the image; F. Threshold adjustments of the image distinguish it from the plate surface and permit analysis of cells as individual particles of defined shape and size.

FIGURE S2

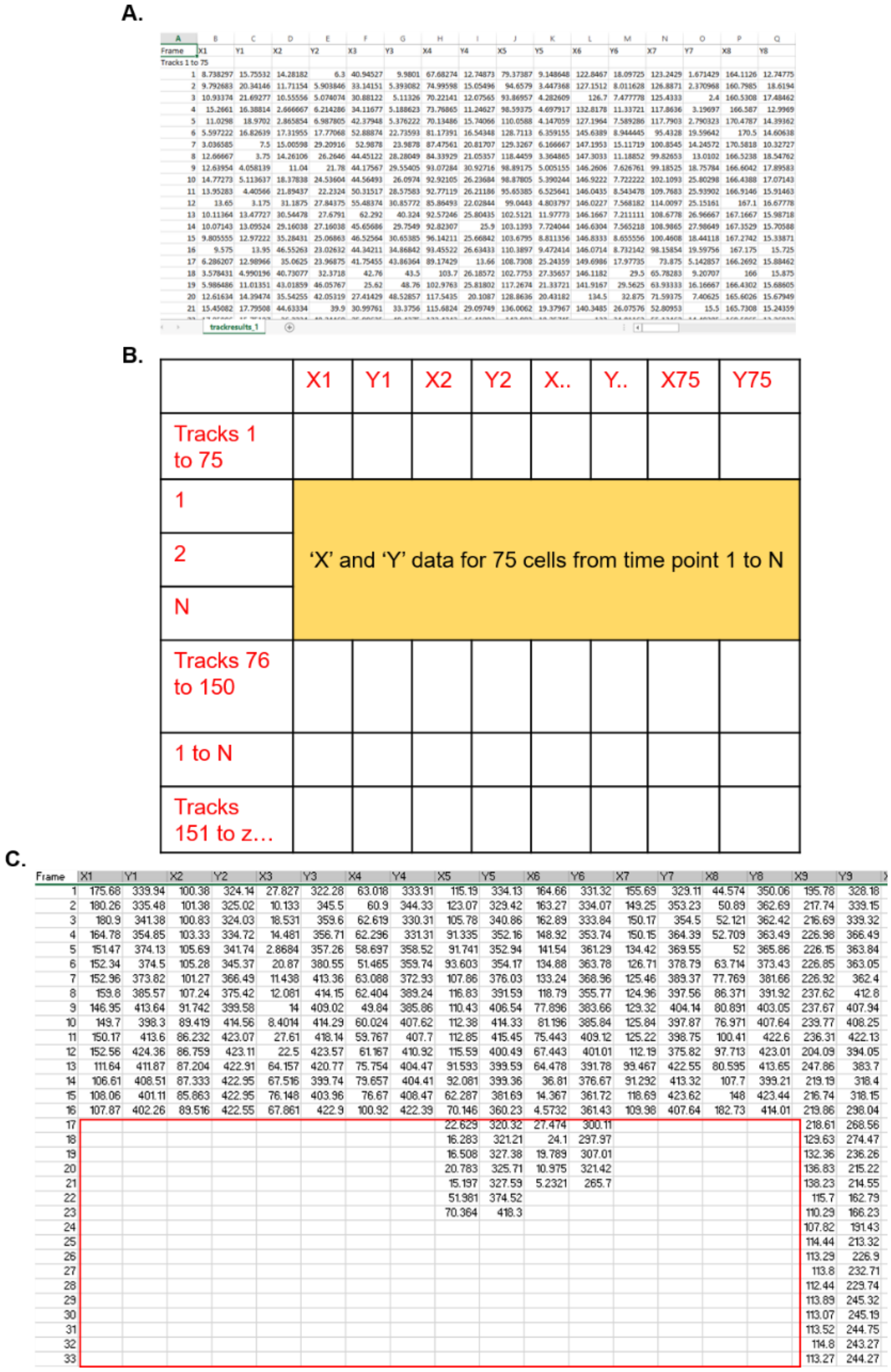

FIGURE S2. Outputs for 'X' – 'Y' co-ordinates. A. Positional data extracted from MTrack2 plugin along with its schematic representation (B); C. Incomplete tracks (marked in red) resulting from cell proliferation or apoptosis are eliminated from further analysis. The entire column containing X and Y positions are deleted from the worksheet.

FIGURE S3

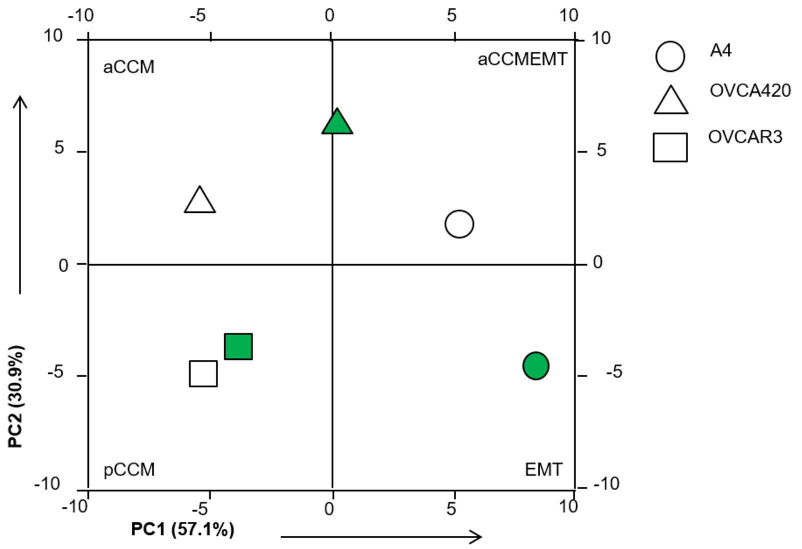

FIGURE S3. TGFβ enhances EMT phenotype in A4 and OVCA420 but not in OVCAR3 cells. PC analysis of time-lapse imaging-based migration data of A4, OVCA420 and OVCAR3 cells, filled (green) and empty shapes indicate presence of TGFβ and absence of serum respectively. Details of statistical analysis are provided in Table S2.

Supplementary Videos

Videos S1 – S3: Time lapse imaging videos depicting the migratory modes of HGSC cell lines (**S1**) A4, (**S2**) OVCA420 and (**S3**) OVCAR3 in the absence of serum. Mitomycin ‘C’ added as a proliferation inhibitor (10µg/mL).

Videos 4 – 6: Time lapse imaging videos depicting the migratory modes of HGSC cell lines (**S4**) A4, (**S5**) OVCA420 and (**S6**) OVCAR3 in the presence of serum.

Videos 7 – 9: Time lapse imaging videos depicting the migratory modes of HGSC cell lines (**S7**) A4, (**S8**) OVCA420 and (**S9**) OVCAR3 in the presence of TGFβ (10ng/mL). Mitomycin ‘C’ added as a proliferation inhibitor (10µg/mL).

Supplementary Table

Table S1. Eigenvalues, percentage of variance captured (eigenvalue normalized by the sum of all eigenvalues) and cumulative percentage of variance captured depicted for PC1 and PC2. Migratory data analysed for cell lines exposed to absence and presence of serum.

Principal Component	Eigenvalues of co-variance	% Variance Captured	Cumulative Variance
PC1	2.69	53.4	53.4
PC2	1.39	28.2	81.6

Table S2. Eigenvalues, percentage of variance captured (eigenvalue normalized by the sum of all eigenvalues) and cumulative percentage of variance captured depicted for PC1 and PC2. Migratory data analysed for cell lines exposed to absence and presence of TGFβ.

Principal Component	Eigenvalues of co-variance	% Variance Captured	Cumulative Variance
PC1	2.81	57.9	57.9
PC2	1.43	30.1	88.0
